# Widespread Exposure to Mosquitoborne California Serogroup Viruses in Caribou, Arctic Fox, Red Fox, and Polar Bears, Canada

**DOI:** 10.3201/eid2901.220154

**Published:** 2023-01

**Authors:** Kayla J. Buhler, Antonia Dibernardo, Nicholas W. Pilfold, N. Jane Harms, Heather Fenton, Suzanne Carriere, Allicia Kelly, Helen Schwantje, Xavier Fernandez Aguilar, Lisa-Marie Leclerc, Geraldine G. Gouin, Nicholas J. Lunn, Evan S. Richardson, David McGeachy, Émilie Bouchard, Adrián Hernández Ortiz, Gustaf Samelius, L. Robbin Lindsay, Michael A. Drebot, Patricia Gaffney, Patrick Leighton, Ray Alisauskas, Emily Jenkins

**Affiliations:** University of Saskatchewan, Saskatoon, Saskatchewan, Canada (K.J. Buhler, É. Bouchard, A. Hernández Ortiz, R. Alisauskas, E. Jenkins);; National Microbiology Laboratory Branch, Winnipeg, Manitoba, Canada (A. Dibernardo, L.R. Lindsay, M.A. Drebot);; San Diego Zoo Wildlife Alliance, Escondido, California, USA (N.W. Pilfold, P. Gaffney);; Government of Yukon, Whitehorse, Yukon, Canada (N.J. Harms);; Ross University School of Veterinary Medicine, Basseterre, St. Kitts and Nevis (H. Fenton);; Government of the Northwest Territories, Yellowknife, Northwest Territories, Canada (H. Fenton, S. Carriere, A. Kelly);; Government of British Columbia, Nanaimo, British Columbia, Canada (H. Schwantje);; University of Calgary, Calgary, Alberta, Canada (X. Fernandez Aguilar);; Government of Nunavut, Kugluktuk, Nunavut, Canada (L.-M. Leclerc);; Makivik Corporation, Kuujjuaq, Québec, Canada (G.G. Gouin);; Environment and Climate Change Canada, Edmonton, Alberta, Canada (N.J. Lunn, D. McGeachy);; Environment and Climate Change Canada, Winnipeg (E.S. Richardson);; Snow Leopard Trust, Seattle, Washington, USA (G. Sameilus);; Université de Montréal, Saint-Hyacinthe, Québec (É. Bouchard, P. Leighton)

**Keywords:** arboviruses, California serogroup viruses, vector-borne infections, viruses, zoonoses, climate change, Jamestown Canyon virus, snowshoe hare virus, caribou, Arctic fox, red fox, polar bear, rodents, Canada

## Abstract

Northern Canada is warming at 3 times the global rate. Thus, changing diversity and distribution of vectors and pathogens is an increasing health concern. California serogroup (CSG) viruses are mosquitoborne arboviruses; wildlife reservoirs in northern ecosystems have not been identified. We detected CSG virus antibodies in 63% (95% CI 58%–67%) of caribou (n = 517), 4% (95% CI 2%–7%) of Arctic foxes (n = 297), 12% (95% CI 6%–21%) of red foxes (n = 77), and 28% (95% CI 24%–33%) of polar bears (n = 377). Sex, age, and summer temperatures were positively associated with polar bear exposure; location, year, and ecotype were associated with caribou exposure. Exposure was highest in boreal caribou and increased from baseline in polar bears after warmer summers. CSG virus exposure of wildlife is linked to climate change in northern Canada and sustained surveillance could be used to measure human health risks.

Annual temperatures in the circumpolar Arctic are rising at 2–3 times the global average, reducing ecologic barriers for arthropod reproduction and fueling shifts in insect diversity and distribution (*1*,*2*). The northward advancement of the tree line and a 50%–60% increase in Arctic precipitation over the past 20 years provide a favorable environment for arthropod emergence (*3*,*4*). Consequently, arboviruses are a growing wildlife and public health concern in the Arctic. Limited information exists on the diversity of arboviruses in Arctic ecosystems, and few studies have identified hosts in sylvatic transmission cycles.

California serogroup (CSG) viruses are antigenically and genetically related emerging vectorborne pathogens of the genus *Orthobunyavirus* that are found throughout North America and are associated with febrile illness and cases of neuroinvasive disease in humans (*5*). Pathogenic strains include La Crosse, Jamestown Canyon (JCV), California encephalitis, snowshoe hare (SSHV), Chatanga, and Inkoo viruses (*6*). Both JCV and SSHV have been identified as causes of arbovirus-associated neurologic diseases in North America (*7*). CSG viruses are transmitted through mosquitoes (*Aedes*, *Culiseta*, and *Anopheles* spp.), maintained by transovarial vector transmission, and circulate in a wide range of vertebrate hosts (*5*,*8*). Since 2006, documented human exposure to CSG viruses has steadily increased in Canada as serologic tests have become available, although infections are still likely underdiagnosed (*5*).

Studies on CSG virus ecology and epidemiology have primarily focused on southern Canada and the contiguous United States. However, recent cases of human exposure in Alaska and the province of Manitoba, Canada have been reported (*9*,*10*), indicating that those viruses exist in northern ecosystems. Human encephalitis in Canada, while rare, has generally been linked to JCV and SSHV serotypes (*5*). Furthermore, we recently detected JCV and SSHV in *Aedes* sp. mosquitoes and biting midges collected in northern Québec (*11*), further confirming circulation of CSG viruses in northern vectors. Potential reservoirs in southern Canada and the United States are cervids for JCV and rodents and lagomorphs for SSHV (*5*). We assessed potential reservoir and sentinel hosts in northern Canada by surveying caribou, rodents and shrews, and carnivores for CSG virus antibodies or RNA across a broad geographic range and identified biologic and ecologic factors associated with exposure.

## Materials and Methods

### Study Area

We collected samples in Yukon, Northwest Territories (NT), Nunavut, Quebec, Manitoba, and British Columbia (BC), Canada (Figure). The study areas comprised tundra, boreal, and mountain ecosystems.

**Figure:**
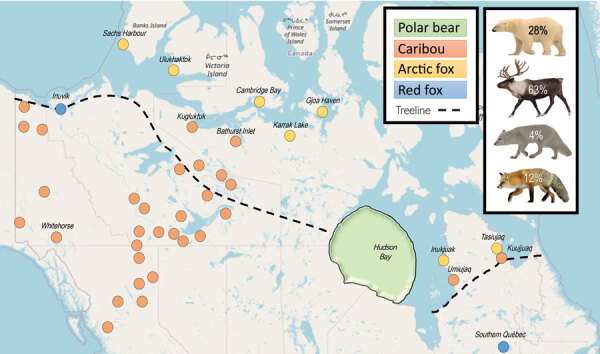
Distribution of animals in study of widespread exposure to mosquitoborne California serogroup viruses in caribou, Arctic fox, red fox, and polar bears, Canada. The green region is the mean on-ice home range of polar bears according to adult female movement (*12*). Locations of caribou include both capture/release and hunter-harvested samples. Dashed line indicates the tree line.

### Sample Collection

We collected blood from hunter-harvested migratory tundra and boreal caribou (*Rangifer tarandus*) in Nunavik (2018, Tasiujaq and Umiujaq, n = 53) and Nunavut (2016, Bathurst Inlet, n = 19). We collected serum samples from caribou live-captured for radio collaring in Yukon (2017–2019, n = 152), BC (2018–2019, n = 20), NT (2010–2019, n = 219) and Nunavut (2019, Kugluktuk, n = 10; 2018, Bathurst Inlet, n = 44) (Figure). We determined sex but not age for caribou.

We collected blood from Arctic and red fox carcasses harvested for fur by licensed trappers in the NT (2018–2019, Inuvik, Sachs Harbour, and Ulukhaktok, n = 72), Nunavut (2019–2021, Cambridge Bay and Gjoa Haven, n = 85), Nunavik (2019–2021, Inukjuak and Tasiujaq, n = 20), and southern Quebec (2016–2017, n = 61). We collected serum samples from Arctic foxes trapped alive at Karrak Lake, Nunavut (2014–2018, n = 108) and Cambridge Bay, Nunavut (2021, n = 28) (*13*). We determined sex of the animals and estimated ages according to a tooth condition index (*14*).

Serum samples were collected from live-captured adult polar bears (n = 377) as part of a long-term study of the western Hudson Bay population during 1986–1989, 1995–1998, and 2015–2017 (*15*,*16*). Sex was determined, and age was estimated by extracting a vestigial premolar tooth and counting cementum annuli (*17*,*18*). 

We collected tissues (instead of blood because of their small size) from rodents and shrews lethally trapped on line transects in the NT during the summers of 2017, 2018, and 2019 (n = 496). We also collected samples at Karrak Lake, Nunavut, during the summers of 2018 and 2019 (n = 9).

### Serology

We stored blood (from carcasses) and serum samples (from live captures) at –20°C until processing. Serologic methods were performed as previously described (*19*). In brief, we detected SSHV and JCV IgM in samples from foxes, caribou, and bears by using a competitive ELISA (cELISA). We measured optical densities at 450 nm, and samples with an inhibition value >30% were considered seropositive. Because this approach was originally developed for serum samples, we compared a positive caribou serum sample diluted in heart blood (1:2) and the same serum sample diluted in blocking buffer (1:2) to identify potential inhibitory effects of whole blood. The dilution in heart blood resulted in 15% loss of inhibition, indicating that whole blood likely underestimates IgM prevalence.

After performing cELISAs, we sent subsets of positive caribou (n = 18) and fox (n = 4) serum samples to the National Microbiology Laboratory in Winnipeg for plaque reduction neutralization tests (PRNTs) to determine exposures to different viruses within the serogroup (*20*). We only conducted differential testing of this subset of animals because of resource limitations arising from the SARS-CoV-2 pandemic. Samples were considered positive for CSG viruses if neutralizing antibody titers were >1:20. A 4-fold increase in titer was used to determine antibody specificity to a single CSG virus versus previous exposures to multiple viruses.

### RNA Extraction and Reverse Transcription PCR for CSG Viruses

We stored tissues from rodents at –20°C until RNA was extracted from a pooled sample of liver, lung, spleen, and kidney for each animal by using the RNeasy Mini Kit (QIAGEN, https://www.qiagen.com). We performed real-time reverse transcription PCR on extracted RNA samples by using the primers CE-NC-F1 (5′-GTGTTTTATGATGTCGCATCA-3′) and CE-NC-R1(5′-CATATACCCTGCATCAGGATCAA-3′) for SSHV and CE-NC-F2 (5′-GTTTTCTATGATGATGCATCC-3′) and CE-NC-R2 (5′-CACAAACCCTGCATCTGGATCAA-3′) for JCV. The probe for both SSHV and JCV was CE-NC (Fam-CAGGTGCAAATGGA-MGB; Integrated DNA Technologies, https://www.idtdna.com). We performed PCR under the following conditions: 50°C for 5 min, 95° for 20 s, then 45 cycles of 95°C for 3 s and 60°C for 30 s. A 20 µL reaction mixture was used containing 5 µL TaqMan Fast Virus 1-Step Master Mix (Thermo Fisher Scientific, https://www.thermofisher.com), 9.4 µL H_2_O, 0.1 µl of each primer (100 µmol/L), 0.2 µL of probe (25 µmol/L), and 5 µL of template. Positive controls were gBlock gene fragments (Integrated DNA Technologies) from the small segment of SSHV and JCV isolates reported in GenBank (accession nos. MK352486.1 and MN135989.1).

### Statistical Analysis

We calculated sample prevalence and 95% CIs by using EpiTools epidemiologic calculators (*21*). We used multiple linear regression to model seropositivity with fixed effect variables 1/0 (positive/negative) as the dependent variables and region, year, age, species, and sex as predictor variables for fox data. We also used multiple linear regression to predict seropositivity (1/0) according to region, year, ecotype, and sex (but not age) for caribou. We classified regions as provinces or territories (BC, Yukon, NT, Nunavut, and Quebec) and ecotypes as migratory tundra, mountain, and boreal. The Leaf River caribou herd in Quebec was classified as migratory tundra caribou during this study, although they are often grouped as woodland forest-tundra caribou.

We also examined co-exposures to CSG viruses and 7 pathogens previously documented in the same polar bears (*18*) by using Pearson χ^2^ tests. Because of the long timeline for polar bear sample collection, we related seroprevalence (1/0) in polar bears to biologic and climatic factors (Table 1) by using binomial (logit link function) generalized linear mixed models and the same constrained set of a priori models for each pathogen as described previously (*18*). In brief, we evaluated sets of biologic and climatic variables separately and identified top factors by using Akaike information criterion corrected for small sample size and weight of the model >0.60. To assess the comparative influence of biologic and climatic factors on CSG virus exposure, we combined top biologic and climatic factors into 1 model and used log-likelihood ratio tests to examine model improvement (reported as χ^2^).

**Table 1:** Covariates used to model the likelihood of California serogroup virus seropositivity in adult polar bears of western Hudson Bay, Canada, 1986–2017, in study of widespread exposure to mosquitoborne California serogroup viruses in caribou, Arctic fox, red fox, and polar bears*

Variables	Range	Description (reference)
Biologic		
Age, y	5–31	Age of polar bear according to tooth histology (*17*)
Sex	1/0#	Field determination with females as reference category
Poor condition†	1/0#	Polar bears rated 1 or 2 on 5-point body condition index (*22*)
Good condition†	1/0#	Polar bears rated 4 or 5 on 5-point body condition index (*22*)
Weight, kg‡	136–602	Calculated weight (*23*) matched to temporal equations for WHB
Conflict§	1/0#	Polar bears captured by Manitoba Conservation in Churchill, MB (*24*) before sample collection
Climatic¶		
Ice free, d	110–152	No. days sea ice concentration was <15% as determined by SSM/I (*25*), within 95% MCP estimate of polar bear home range (*12*)
Summer temperature, °C	7.8–10.8	Mean air temperature, June–September, measured at Churchill airport, MB (*26*)
Summer precipitation, mm	169.0–310.6	Total precipitation, June–September, measured at Churchill airport, MB (*26*)
Winter temperature, °C	–30.0 to –24.9	Mean minimum air temperature, December–March, measured at Churchill airport, MB (*26*)
Annual temperature, °C	–7.4 to –5.2	Mean annual air temperature measured at Churchill airport, MB (*26*)
Annual precipitation, mm	344.7–507.8	Total annual precipitation measured at Churchill Airport, MB (*26*)

*This table was published previously (*18*). MB, Manitoba; MCP, minimum complex polygon; SSM/I, special sensor microwave/imager; WHB, western Hudson Bay. †The 5-point body condition index was dummy-coded with an average score of 3 forming the reference category. ‡Mean weight centered within sex before modeling. §Bears with a history of capture as part of the Polar Bear Alert Program prior to sampling. ¶All climate variables measured the year before serum sample collection. #Multiple linear regression was used to model seropositivity with fixed effect variables 1/0 (positive/negative) as dependent variables.

We performed analyses by using SPSS Statistics 28 (IBM, https://www.ibm.com) for caribou and foxes and R software version 3.3.3 (The R Project for Statistical Computing, https://www.r-project.org) for polar bears. We reported all variances with 95% CIs, and α was set to 0.05 for significance. We considered all animals sampled multiple times during the study (bears, n = 40; Arctic foxes, n = 12; Caribou; n = 52) positive if a single blood sample tested positive. We did not include subsequent results from positive animals in the analyses because duration of virus antibody production is not well understood.

## Results

### Prevalence

Mean seroprevalence was 63% (95% CI 58%–67%, n = 517) for caribou, 4% (95% CI 2%–7%, n = 297) for Arctic foxes, 12% (95% CI 6%–21%, n = 77) for red foxes, and 28% (95% CI 24%–33%, n = 377) for polar bears (Table 2). The prevalence for bears varied significantly between time periods (χ^2^ = 9.98, degrees of freedom [d.f.] = 2, p = 0.007); a significant increase in positive cases was observed between the mid-1980s and mid-1990s (χ^2^ = 9.78, d.f. = 1, p = 0.002). Seropositivity in the mid-2010s did not significantly differ from either the mid-1980s (χ^2^ = 1.55, d.f. = 1, p = 0.213) or mid-1990s (χ^2^ = 2.71, d.f. = 1, p = 0.100) (Table 3). Polar bears sampled repeatedly (3 during 1995–1998 and 2 during 2015–2017) had positive titers that decreased below the cELISA threshold in subsequent sampling. Three of those bears were sampled again 1 year after initial positive samples, indicating that virus antibodies were short-lived (inhibition values were 38%, 45%, and 94% the year before). The other 2 bears were sampled 18–19 years after initial positive samples.

**Table 2:** Prevalence of California serogroup viruses in wildlife in Canada, 2017, in study of widespread exposure to mosquitoborne California serogroup viruses in caribou, Arctic fox, red fox, and polar bears*

Species	Sample size	Test	Total prevalence, % (no./total)	Location	Regional prevalence, % (95% CI) (no./total)	Prevalence, serum, % (no./total)	Prevalence, whole blood, % (no./total)
Caribou	517	cELISA	63 (324/517)	BC	45 (26–66) (9/20)	45	NA
				Yukon	45 (37–53) (68/152)	45	NA
				NT	83 (78–88) (182/219)	83	NA
				Nunavut	80 (69–87) (58/73)	91 (49/54)	47 (9/19)
				Nunavik	13 (7–25) (7/53)	NA	13
Arctic fox	297	cELISA	4 (11/297)	NT	0 (0–6) (0/66)	NA	0
				Nunavut	4 (2–7) (8/221)	5 (7/136)	1 (1/85)
				Nunavik	30 (11–60) (3/10)	NA	30
Red fox	77	cELISA	12 (9/77)	NT	0 (0–39) (0/6)	NA	0
				Nunavik	20 (6–51) (2/10)	NA	20
				South QC	12 (6–22) (7/61)	NA	12
Polar bear	377	cELISA	28 (107/377)	Manitoba	28 (24–33)	28	NA
Red-backed vole	349	qPCR	0	NT	0 (0–1)	NA	NA
Meadow vole	20	qPCR	0	NT	0 (0–16)	NA	NA
Deer mouse	68	qPCR	0	NT	0 (0–5)	NA	NA
Collared lemming	9	qPCR	0	Nunavut	0 (0–30)	NA	NA
Shrew, unidentified	59	qPCR	0	NT	0 (0–6)	NA	NA

*BC, British Columbia; MB, Manitoba; NA, not applicable; NT, Northwest Territories; QC, Québec; qPCR; quantitative PCR; cELISA, competitive ELISA.

**Table 3:** Seroprevalence of California serogroup viruses in the western Hudson Bay polar bear population during 3 periods in study of widespread exposure to mosquitoborne California serogroup viruses in caribou, Arctic fox, red fox, and polar bears, Canada*

Years	Sample size	Prevalence, % (95% CI)	No. males	Prevalence, % (no.)	No. females	Prevalence, % (no.)
1986–1989	142	21 (13–26)	67	18 (12)	70	23 (16)
1995–1998	149	36 (28–44)	73	23 (17)	76	47 (36)
2015–2017	100	27 (19–36)	47	17 (8)	53	36 (19)

*Repeat samples from bears were counted individually if bears were sampled between periods. Only one sampling was counted if bears were sampled multiple times within the same period. Sex was not determined for 5 bears.

Estimated seroprevalence varied between regions for caribou and foxes; the highest prevalence was observed in the NT (83%, n = 219) and Nunavut (80%, n = 73) for caribou and in Nunavik for red foxes (20%, n = 10) and Arctic foxes (30%, n = 10) (Table 2). Boreal caribou (87%, n = 172) were exposed more often than migratory tundra (48%; n = 243) or mountain caribou (59%, n = 87) (Table 4). By PRNT, 18 positive cELISA samples from caribou in the NT had a positive titer >1:20 for JCV. We observed an SSHV titer of 1:40 in 4 caribou, 2 of which had a JCV titer of 1:160 and 1:320, indicating exposure to JCV. Furthermore, PRNT of 4 positive fox samples (3 from Nunavut and 1 from Quebec) indicated exposure to JCV.

**Table 4:** Prevalence of California serogroup viruses within caribou ecotypes and herds/study areas across Canada in study of widespread exposure to mosquitoborne California serogroup viruses in caribou, Arctic fox, red fox, and polar bears*

Ecotype	Prevalence, % (95% CI) (no./total)	Herd or study area	Capture location	Prevalence, % (95% CI) (no./total)
Migratory tundra caribou	48 (42–54) (116/243)	Beverly	NT	83 (55–95) (10/12)
		Bluenose East	NT	60 (36–80) (9/15)
		Bathurst	NT	77 (50–92) (10/13)
		Dolphin and Union	Nunavut	80 (69–87) (58/73)
		Porcupine	Yukon	24 (15–35) (17/72)
		Forty Mile	Yukon	100 (57–100) (5/5)
		Leaf River	Nunavik, QC	13 (7–25) (7/53)
Mountain woodland caribou	59 (48–68) (51/87)	Heart River	Yukon	78 (58–90) (18/23)
		Ibex	Yukon	38 (14–69) (3/8)
		Clear Creek	Yukon	70 (48–86) (14/20)
		Carcross	Yukon	43 (25–64) (9/21)
		Tay River	Yukon	50 (10–91) (1/2)
		Laberge	Yukon	100 (21–100) (1/1)
		Pink Mountain	BC	67 (21–94) (2/3)
		Muskwa	BC	24 (5–70) (1/4)
		Kennedy Siding	BC	50 (10–91) (1/2)
		Itcha-Ilgachuz	BC	0 (0–79) (0/1)
		Chase	BC	0 (0–79) (0/1)
		Quinette	BC	100 (21–100) (1/1)
Boreal woodland caribou	87 (81–91) (149/172)	Chinchaga	BC	50 (10–91) (1/2)
		Snake-Sahtaneh	BC	100 (21–100) (1/1)
		Maxhamish	BC	0 (0–79) (0/1)
		Calendar	BC	100 (21–100) (1/1)
		North Deh Cho	NT	92 (78–97) (33/36)
		South Deh Cho	NT	89 (74–95) (31/35)
		Pine Point-Buffalo Lake	NT	97 (85–99) (34/35)
		Hay River Lowlands	NT	78 (61–89) (25/32)
		Mackenzie	NT	79 (62–90) (23/29)

*Ecotype data were not available for 13 caribou; herd/study area data were not available for 15 caribou. BC, British Columbia; NT, Northwest Territories; QC, Québec.

Of the caribou that were repeatedly sampled during 2016–2018 (n = 52), 3 animals had titers that dropped below the cELISA cutoff value between winter and the following spring. Conversely, 3 animals seroconverted during the same time frame (inhibition values between 31%–43%). We did not detect viral RNA by PCR in samples from 349 red backed voles (*Myodes rutilus*), 20 meadow voles (*Microtus pennsylvanicus*), 68 deer mice (*Peromyscus maniculatus*), 9 collared lemmings (*Dicrostonyx groenlandicus*), and 59 shrews (species unidentified).

### Biologic, Ecologic, and Climatic Factors

We did not detect substantial co-occurrence between CSG viruses and 7 other pathogens previously examined in the same polar bears (*18*). Both biologic and climatic factors influenced polar bear exposure to CSG viruses. Adult female polar bears were 2.6 (95% CI 1.6–4.2) times more likely to be seropositive than adult male polar bears. Age was negatively correlated with seropositivity, although the 95% CI included zero (β = −0.04, 95% CI –0.04 to 0.0). Polar bears previously captured in the town of Churchill, Manitoba, were 3.4 (95% CI 1.8–6.4) times less likely to be seropositive for CSG viruses. Summer temperature in the preceding year (corrected Akaike information criterion, weight of model = 0.97) was a top climatic factor in the model, and warmer summer air temperatures were positively correlated with polar bear exposure to CSG viruses (β = 0.78, 95% CI 0.47–1.09). Inclusion of biologic and climatic factors in the same model significantly improved model fit (χ*^2^* = 29.0, d.f. = 3, p<0.001) (Appendix Table 1).

Biologic factors did not influence fox exposure to CSG viruses; however, location was significantly associated with seroprevalence (β = −0.2, p<0.05) and was highest in foxes in the eastern Arctic (*R^2^* = 0.06, d.f. = 5, p<0.05). For caribou, location was also significantly associated with exposure (β = −0.6, p<0.001); the highest prevalence was observed in Nunavut and NT. In addition, ecotype (β = 0.3, p<0.001) and year (β = −0.2, p<0.001) were significant variables in the model (*R^2^* = 0.22, d.f. = 4, p<0.001); the highest exposures occurred in 2010 (94%) and 2012 (93%) in the boreal woodland ecotype (Table 4).

## Discussion

This study demonstrates widespread exposure to mosquitoborne viruses in wildlife across northern Canada. Caribou were most often exposed to CSG viruses (likely JCV) with seroprevalence >80% in the NT and Nunavut. The high prevalence, along with identification of cervids as reservoir hosts in temperate regions, suggests that caribou might serve as reservoirs and sentinels for JCV (*27*). Caribou congregate in herds and are particularly vulnerable to arthropod bites during calving when they are sedentary (*28*). Thus, we expected higher rates of CSG virus exposure in caribou than in polar bears that spend a considerable amount of time on sea ice or foxes that have a smaller body size and are nocturnally active (*29**,**30*). Sampling location (province/territory) was associated with exposure, and high seroprevalence in caribou in central and western Arctic regions contrasted with 13% seroprevalence in the eastern Arctic (Nunavik, Quebec). This result reflects a difference in virus prevalence, although the use of whole blood from harvested animals in the eastern Arctic likely underestimated seroprevalence, especially in the migratory tundra ecotype. Noticeable differences in prevalence were also observed between whole blood and serum samples from caribou in Nunavut (Table 2), indicating that whole blood is likely not an ideal sample for the cELISA.

Canada’s changing climate might play a role in CSG virus seroprevalence. The western Arctic region in Canada is warming more rapidly than the eastern Arctic region of Canada and the rest of the world (*2*). Warming temperatures have been linked to changes in mosquito diversity, density, distribution, and host-seeking behaviors (*31**,**32*). For example, rising temperatures can increase mosquito development and survival and bring mosquitoes into phenologic synchrony with caribou, providing opportunities for pathogen transmission (*31*). Increases in precipitation might also influence regional differences in vector abundance and competence. Normalized precipitation increased 30% from 1948 to 2012 in the Arctic region of Canada (*33*), especially in Nunavut, thereby increasing the abundance of larval development sites for mosquito vectors (*2**,**34*). Sampling year also influenced caribou exposure. Therefore, future long-term studies should investigate associations between climate and temporal patterns of exposure in caribou, as we did for polar bears.

Ecotype was the final factor that was associated with caribou exposure. Boreal caribou had greater exposure than those in other ecotypes (87%; Table 4). Boreal caribou remain in treed environments year-round, whereas tundra and mountain caribou migrate to tundra and alpine habitats during the summer months (*35*,*36*). Warmer temperatures at lower altitudes and lower windspeeds in treed environments might increase exposure to insect bites (*37*). Climate change has been linked to the northward advancement of the tree line, which might increase exposure to CSG viruses for caribou populations in the future (*4*). These factors, along with differences in the distribution and diversity of mosquito species and their vector competence, might all contribute to observed variations in seroprevalence among caribou and might also correlate with risk for human exposure.

In foxes, overall CSG virus seroprevalence was much lower than for caribou, likely because of lower exposure to mosquitoes (smaller body size, nocturnal activity) (*30*). Region was associated with exposure in foxes. However, contrary to the results from caribou, the highest seroprevalence for foxes was in northern Quebec (30% for Arctic fox and 20% for red fox). This result might reflect a difference in viral serotype in foxes. Caution is warranted when interpreting these results because the sample size in this region was small (n = 10 for each species) and only samples from 4 foxes were successfully tested by using PRNTs, which revealed exposure to JCV. Increasing the number of fox samples tested with cELISA and PRNT would help identify what CSG viruses are present in northern Quebec.

Archived serum samples from western Hudson Bay polar bears provided a unique opportunity to monitor changes in exposure to CSG viruses and other pathogens (*18*) over time in one of the most rapidly warming Arctic regions in Canada. Exposure to CSG viruses in polar bears increased between 1986–1989 and 1995–1998 but did not continue to increase during 2015–2017 (Table 3). We found a strong positive association between air temperatures in the previous summer and virus exposure. Warmer air temperatures during summer when bears are on land likely increased the abundance of mosquitoes and bite exposures, especially in peatland ecosystems that are not moisture limited, overwhelming the influence of other climatic factors on CSG virus exposure. Summer air temperature and ice-free days did not increase from 1995–1998 to 2015–2017 (Appendix Table 2), which might explain the lack of continued increase in exposure to CSG viruses. However, because sea ice breakup in western Hudson Bay has been occurring ≈5–6 days earlier per decade (*15*,*29*) and temperatures continue to rise, polar bear exposures to vectorborne pathogens, including CSG viruses, will likely increase.

Summer segregation of polar bears by age and sex might partly explain why female and younger adult bears had higher CSG virus exposure. Hudson Bay is ice-free during the summer and fall, forcing polar bears onshore for 3–4 months; pregnant females are forced onshore for 8 months (*38*–*40*). While onshore, adult males are typically found in drier coastal areas, whereas adult females with cubs and pregnant females travel inland (*39*). The inland area consists of peatlands that are underlain by continuous permafrost resulting in poor drainage and extensive bogs and fens (*41*,*42*). Dens are constructed in peat deposits near water sources (*41*–*44*). Thus, proximity to stagnant water likely accounts for increased exposure of females and young bears to mosquito bites. Our study design limited the analysis to adult polar bears, and the age effect might have been more pronounced with the inclusion of younger animals.

Polar bears that were captured in Churchill were less likely to be exposed to CSG viruses, which is congruent with patterns of exposure to *Francisella tularensis* previously described (*18*). Similar to CSG viruses, the life cycle of *F. tularensis* involves transmission by biting insects (*45*). Churchill is on the Hudson Bay coast, and polar bears previously captured in town might be more likely to inhabit coastal areas that have reduced exposure to biting insects than polar bears found farther inland. These results suggest that persons in coastal regions of the Arctic have lower risk for arboviral exposure than those who live or travel inland.

Rodents and lagomorphs are theorized reservoirs for SSHV (*5*). However, all rodent samples tested during this study were negative for SSHV RNA, possibly caused by the short viremia duration typically associated with arboviral infections or by sample storage. For example, white-tailed deer (*Odocoileus virginianus*) had detectable JCV in the blood for only 2–4 days after inoculation (*27*). Thus, antibody rather than virus detection might be more practical for CSG virus surveillance, and hosts with larger blood volume (such as hares) might be better suited as sentinels for SSHV surveillance. However, serologic methods also introduce challenges. Results from repeatedly sampled caribou and polar bears suggest that antibodies might be relatively short-lived. In addition, 3 caribou seroconverted over winter, which suggests that false positives are possible.

All caribou samples from the NT tested by using PRNT had neutralizing antibodies against JCV, which was expected because white-tailed deer have been suggested as reservoir hosts for JCV in the United States and Canada (*46*,*47*). In Quebec, areas with moderate densities of white-tailed deer were associated with greater risk for human JCV seropositivity (*48*). Although exposure to JCV was expected, 4 caribou from the NT also had antibodies against SSHV, suggesting serologic cross-reactivity or exposure to both viruses.

Climate change, along with a deep cultural relationship between Indigenous persons and wildlife, suggests that northern Canada is an ideal location to study the effects of environmental variability on diseases that affect both human and animal health (*49*). This study demonstrated widespread distribution and regional differences in exposure to CSG viruses in wildlife of northern Canada across multiple ecosystems, highlighting the benefit of monitoring wildlife as sentinels for human disease risk. We identified high CSG virus seroprevalence in caribou populations, some of which are declining across northern Canada, emphasizing the need to determine whether caribou are reservoir hosts and whether JCV affects health and fecundity of these animals. Our finding of increased CSG virus seroprevalence in polar bears over time demonstrates the utility of comparing prevalence of climate sensitive diseases against baseline values for species known to be affected by rapid climate change (*18*,*50*). We identified summer air temperature as a key factor influencing polar bear exposure to CSG viruses, suggesting that infections will likely become more prevalent as the climate continues to change. Our study provides preliminary data for future surveillance of mosquitoborne viruses and highlights the need for continued studies to decipher the transmission dynamics of vectorborne diseases in regions experiencing substantial climate change. Future sustained surveillance of CSG and other arboviruses would provide additional information to measure health risks for humans and wildlife of conservation significance.

Appendix Additional information for widespread exposure to mosquitoborne California serogroup viruses in caribou, Arctic fox, red fox, and polar bears, Canada.
