# Supplementary material for: Widespread Exposure to Mosquitoborne California Serogroup Viruses in Caribou, Arctic Fox, Red Fox, and Polar Bears, Canada
Source: Emerg Infect Dis. 2023 Jan;29(1):54–63. doi: 10.3201/eid2901.220154 (PMC9796188; doi:10.3201/eid2901.220154)
Supplement: Appendix — Additional information for widespread exposure to mosquitoborne California serogroup viruses in caribou, Arctic fox, red fox, and polar bears, Canada. [file 22-0154-Techapp-s1.pdf]

Article DOI: https://doi.org/10.3201/eid2901.220154

Widespread Exposure to Mosquitoborne California Serogroup Viruses in Caribou, Arctic Fox, Red Fox, and Polar Bears, Canada

Appendix

Appendix Table 1. Comparison of model fit for top biologic and climatic factors linked to seroprevalence of California serogroup viruses in adult polar bears of western Hudson Bay, Canada, 1986–2017 in study of widespread exposure to mosquitoborne California serogroup viruses in caribou, Arctic fox, red fox, and polar bears*

Rank	Type	Model	k	LL	AIC_C	ΔAIC_C
1	Combined	(1|BearID) + sex + age + conflict† + STemp	6	−207.90	427.98	0.00
2	Biologic	(1|BearID) + sex + age +conflict	5	−222.40	463.14	35.16
3	Climatic	(1|BearID) + STemp	3	−229.00	464.04	36.07
4	Null	(1|BearID)	2	−241.50	487.06	59.09

*Models were fitted by using a binomial logit-link generalized linear mixed model with individual Bear ID (1|BearID) as a random effect to account for repeat animals. AIC_c, Akaike's information criterion for small samples; k, penalty multiplier for model complexity; LL, log-likelihood; STemp, summer temperature.
†Bears with a history of capture as part of the Polar Bear Alert Program prior to sampling.

Appendix Table 2. Mean values for climatic variables used in binomial logit-link generalized linear mixed models for each period of polar bear exposure to California serogroup viruses in the Hudson Bay, Canada, 1986–2017, in study of widespread exposure to mosquitoborne California serogroup viruses in caribou, Arctic fox, red fox, and polar bears*

Variables†	1986–1989	1995–1998	2015–2017
Ice free, d	114 ± 6	141 ± 16	145 ± 7
Summer temperature, °C	9.1 ± 0.9	10.5 ± 0.6	10.3 ± 0.9
Summer precipitation, mm	212.5 ± 49.5	274.1 ± 42.4	226.0 ± 16.0
Winter minimum temperature, °C	−27.5 ± 2.0	−27.2 ± 1.9	−25.9 ± 1.3
Annual temperature, °C	−7.0 ± 1.1	−6.3 ± 1.2	−5.7 ± 1.3
Annual precipitation, mm	431.7 ± 56.9	458.0 ± 45.4	366.4 ± 38.3

*This table was published previously (*18*).
†Values are mean ±SD.
